# Small bowel lymphoma presenting with frequent perforation: a single center experience focusing on the monomorphic epitheliotropic intestinal T-cell lymphoma

**DOI:** 10.1186/s12876-026-04641-8

**Published:** 2026-01-23

**Authors:** Wan-Chih Yeh, Ming-Lun Han, Chia-Hung Tu, Chien-Chuan Chen, Tsu-Yao Cheng

**Affiliations:** 1 Department of Internal Medicine, Division of Gastroenterology, National Taiwan University Hospital Yunlin Branch, Douliu, Taiwan; 2 Department of Internal Medicine, Division of Gastroenterology, National Taiwan University Hospital, Taipei, Taiwan; 3 Department of Laboratory Medicine, National Taiwan University Hospital, 7, Chung Shan South Road, Taipei, 100225 Taiwan; 4 Department of Laboratory Medicine, National Taiwan University College of Medicine, Taipei, Taiwan

**Keywords:** Monomorphic epitheliotropic intestinal T-cell lymphoma, Small bowel cancer, Gastrointestinal lymphoma, Perforation, Enteroscopy

## Abstract

**Background:**

Small bowel malignancies are rare and initial symptoms are generally vague and non-specific. Monomorphic epitheliotropic intestinal T-cell lymphoma (MEITL) is a primary lymphoma of the gastrointestinal tract typically localizing to the small bowel. We attempted to analyze small bowel lymphoma patients in a tertiary care center focusing on the current diagnostic and treatment strategies of MEITL.

**Methods:**

We reviewed patients with histopathology diagnosis of small bowel lymphoma from January 1998 to December 2018. Survival analysis was performed using the Kaplan–Meier method.

**Results:**

There were 140 lymphoma patients with small bowel involvement including 13 MEITL patients (9%). The incidence of small bowel perforation was much higher in MEITL patients than the non-MEITL small bowel lymphoma patients (46% versus 8%, *p* < .001). MEITL was significantly associated with weight loss, chronic diarrhea, elevated white blood cell counts, and low albumin levels. Most MEITL patients had jejunal involvement with diffuse bowel wall thickening and luminal narrowing in imaging studies. Enteroscopic findings in MEITL were typically characterized by edematous mucosal thickening and multifocal shallow ulcerations, frequently observed alongside a mosaic mucosal pattern or diffuse erosions. Most MEITL patients had received chemotherapy, and two patients received chemotherapy along with autologous stem cell transplantation. The median survival of MEITL was 8.0 months, and was significantly associated with age, performance, perforation status, and anthracycline chemotherapy.

**Conclusions:**

As a rare lymphoma with poor prognosis, MEITL should be considered in Asian patients with jejunal lesions presenting with weight loss, chronic diarrhea, bowel perforation, and characteristic imaging/endoscopic features. Intensive chemotherapy followed by autologous stem cell transplantation may provide a better outcome.

## Introduction

Primary small bowel malignancies are very rare though the small bowel accounts for more than 90% of the mucosal surface of the whole digestive tract [1]. The initial symptoms of small bowel tumors are generally vague and non-specific. Intermittent abdominal pain, occult bleeding with anemia, and obstruction may develop in patients with small bowel tumors. Furthermore, weight loss is a common clinical manifestation of small bowel malignancies [2]. The diagnosis of small bowel malignancies can be delayed up to two years due to failure to order appropriate diagnostic tests and misinterpretation of imaging studies [3]. Recently, the clinical use of capsule endoscopy (CE) and device-assisted enteroscopy (DAE) with biopsy has increased in Asian countries, including Taiwan. This trend is driven in part by the lower procedural costs relative to Western nations [4]. These advanced techniques represent a significant opportunity for the earlier detection and diagnosis of small bowel malignancies.

Monomorphic epitheliotropic intestinal T-cell lymphoma (MEITL), formerly known as type II enteropathy associated T-cell lymphoma (EATL) but renamed in the 2016 WHO classification, is a rare primary lymphoma of the gastrointestinal tract typically localizing to the small bowel [5]. MEITL predominating in Asian countries shows no association with celiac disease, while EATL/type I EATL predominating in northern Europe is closely linked to celiac disease [6]. In comparison with EATL, MEITL may similarly present with an acute abdomen and intestinal perforation, but fewer MEITL patients present with prior chronic gastrointestinal symptoms [7].

This article detailed a tertiary care center’s experience with small bowel lymphomas, with a specific focus on the clinical profile of MEITL. We aimed to heighten clinical awareness among gastroenterologists regarding MEITL and to underscore the importance of specialized diagnostic imaging, enteroscopy, and current therapeutic strategies.

## Methods

### Study population

We reviewed consecutive patients with histopathology diagnosis of lymphoma in the small bowel at a tertiary care center, National Taiwan University Hospital, over a 21-year period between January 1998 and December 2018. Clinical data were collected from both physical and electronic patient records. All data were analyzed retrospectively. Inclusion criteria consisted of patients aged 20 or older and diagnosed with small bowel lymphoma. Patients with underlying two or more malignancies were excluded from the study. Cases were classified as MEITL or EATL according to the WHO criteria. MEITL was defined by a monomorphic proliferation of neoplastic lymphocytes (small- to medium-sized cells) with prominent epitheliotropism, the absence of celiac disease, and a characteristic CD8+/CD56 + immunophenotype. EATL was diagnosed by a polymorphic lymphoid infiltrate (medium- to large-sized cells) with an inflammatory background, an association with celiac disease (clinically or histologically), and an immunohistochemical profile mainly CD8-/CD56- [5, 7].

### Statistical analysis

Survival analysis was performed by the Kaplan–Meier method. Categorical data were analyzed using Pearson chi-square test, or Fisher’s exact test in cases with small samples. Mann-Whitney U test was used to compare two medians. Data were analyzed with the IBM SPSS Statistics for Windows, version 22 (IBM Corp., Armonk, N.Y., USA). Statistical significance was set at the standard 5% level.

## Results

### Patients’ demographics and clinical characteristics

**Fig. 1:**
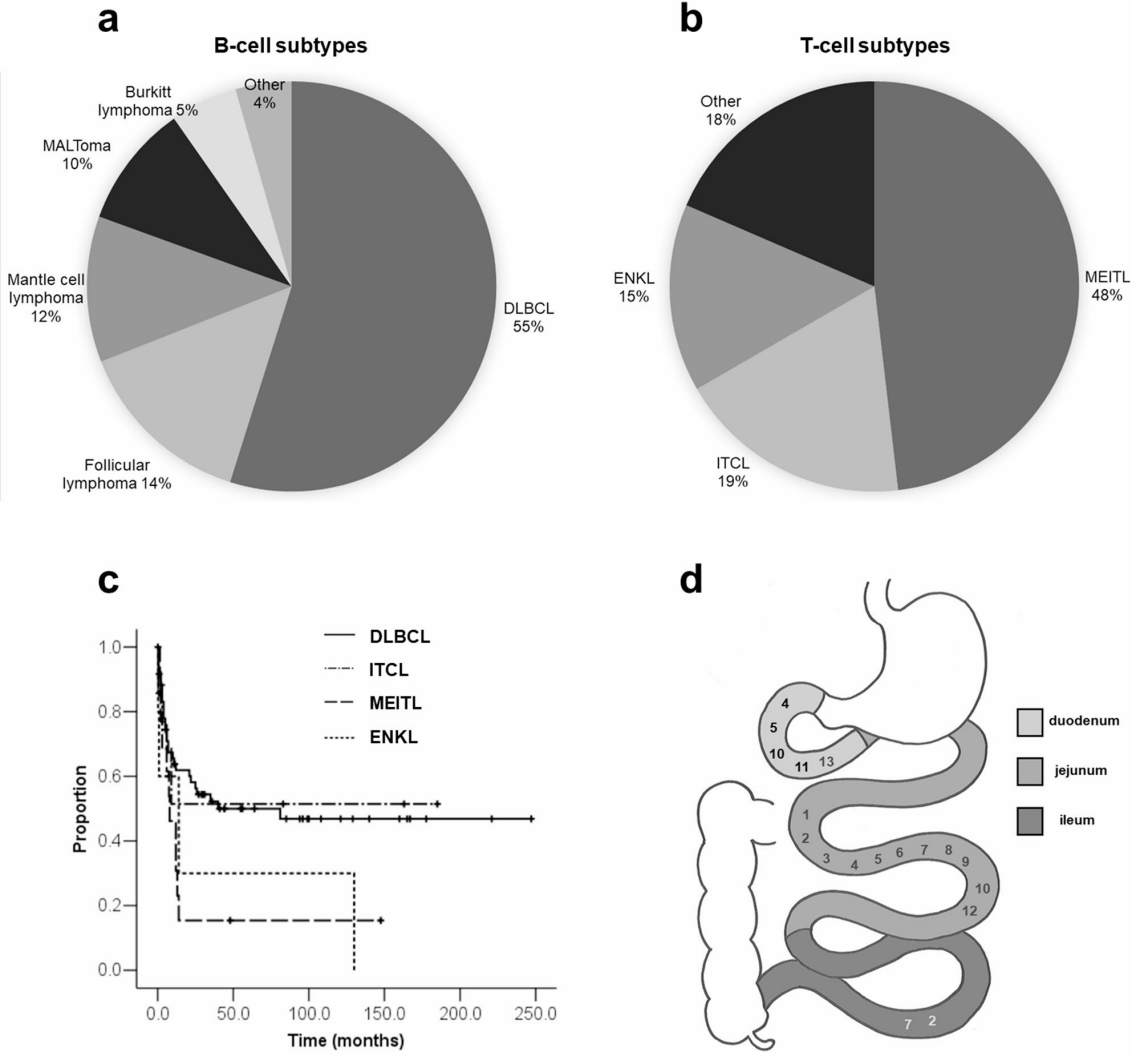
Characteristics of MEITL. Distribution of histologic subtypes of (**a**) B-cell lymphomas and (**b**) T-cell lymphomas with small bowel involvement. **c** Kaplan-Meier analysis of overall survival. **d** Anatomical distribution of MEITL in the small bowel

There were 140 lymphoma patients with small bowel involvement. In our series, most small bowel lymphoma patients were B-cell lymphomas (113/140, 81%). Diffuse large B-cell lymphoma (DLBCL) and follicular lymphoma were the most prevalent small bowel B-cell lymphomas, accounting for 55% and 14% of cases, respectively, followed by mantle cell lymphoma (12%) and mucosa-associated lymphoid tissue lymphoma (MALToma) (10%) (Fig. 1a). In contrast, T-cell lymphomas were less common, occurring in only 19% of cases (27/140). MEITL, Intestinal T-cell lymphoma (ITCL), and extranodal NK/T-cell lymphoma (ENKL) were the primary subtypes identified, accounting for 48%, 19%, and 15%, respectively. (Fig. 1b).

The most common symptom in all small bowel lymphoma patients was abdominal pain. Compared to the non-MEITL small bowel lymphoma cohort, patients with MEITL presented with significantly higher rates of weight loss and chronic diarrhea. Anatomically, MEITL was characterized by more frequent jejunal involvement and a lower incidence of ileal disease. Laboratory analysis revealed significantly elevated white blood cell (WBC) counts and lower serum albumin levels in the MEITL group. Bone marrow involvement was rare, occurring in only two patients (15%). At the time of diagnosis, approximately one-third of patients (31%) presented with stage I/II disease. Small bowel perforation was the most prevalent complication, with a significantly higher incidence in MEITL patients compared to the non-MEITL group (46% versus 8%, *p* < .001) (Table 1). Perforation sites involved the jejunum exclusively (*n* = 3), both the jejunum and ileum (*n* = 2), and the duodenojejunal junction (*n* = 1). All six patients underwent small bowel resection followed by primary anastomosis. Within the MEITL cohort, all perforation events occurred at the time of initial diagnosis. Similarly, most non-MEITL perforations were identified at diagnosis, with the exception of one DLBCL case that presented as a complication during chemotherapy. Under further subgroup analysis, the odds of perforation in MEITL patients were 25-fold, 6.7-fold, and 25-fold higher than those in patients with follicular lymphoma (*P* = .004), DLBCL (*P* = .008), and mantle cell lymphoma (*P* = .015). Perforation was significantly more frequent in MEITL than in either indolent (e.g., follicular) or other aggressive (e.g., DLBCL, mantle cell) lymphoma subtypes (Table 2).

**Table 1:** Clinical features of lymphomas with small bowel involvement

	MEITL (*N* = 13)	Non-MEITL lymphoma (*N* = 127)	*P* value
Age (years)			
Median age	58 (26–79)	57 (4–92)	0.760 ^*c*^
Gender			
Male	10 (77%)	75 (59%)	0.209 ^*a*^
Female	3 (23%)	52 (41%)	
Primary symptoms while diagnosed			
Abdominal pain	8 (62%)	56 (44%)	0.229 ^*a*^
Weight loss	7 (54%)	26 (20%)	0.007 ^*b*^ *
Chronic diarrhea	5 (38%)	6 (5%)	0.001 ^*b*^ *
Site			
Duodenum	4 (31%)	51 (40%)	0.509 ^*a*^
Jejunum	11 (85%)	24 (19%)	< 0.001 ^*b*^ *
Ileum	2 (15%)	59 (46%)	0.031^*a*^ *
Laboratory data (median)			
WBC (K/µL)	9.74	6.98	0.002 ^*c*^ *
Hb (g/dL)	11.7	11.0	0.264 ^*c*^
PLT (K/µL)	335	248	0.051 ^*c*^
LDH (U/L)	379	391	0.557 ^*c*^
Albumin (g/dL)	2.60	3.65	< 0.001 ^*c*^ *
Stage			
I/II	4 (31%)	45 (35%)	> 0.999 ^*b*^
III/IV	9 (69%)	82 (65%)	
IPI score			
Mean IPI score	3	2.37	0.573 ^*c*^
Complications			
Perforation	6 (46%)	10 (8%)	< 0.001 ^*a*^ *
GI tract bleeding	0 (0%)	21 (17%)	0.217 ^*b*^
Prognosis			
Alive	2 (15%)	53 (42%)	0.064 ^*a*^

^*a*^ Pearson chi-square test

^*b*^ Fisher’s exact test

^c^ Mann-Whitney U test

* *P* < 0.05

**Table 2:** Frequency of perforation in MEITL compared to other lymphoma subtypes

		Perforation, *n* (%)	Odds ratio (95% CI)	*P* value	
MEITL (*N* = 13)		6 (46%)	1.00		-
Burkitt lymphoma (*N* = 6)		1 (17%)	0.23 (0.02–2.59)		0.333 ^*a*^
ENKL (*N* = 4)		1 (25%)	0.39 (0.03–4.80)		0.603 ^*a*^
ITCL (*N* = 5)		0 (0)	0.11 (0.005–2.28) ^*b*^		0.114 ^*a*^
DLBCL (*N* = 62)		7 (11%)	0.15 (0.04–0.57)		0.008 ^*a*^ *
Mantle cell lymphoma (*N* = 13)		0 (0)	0.04 (0.002–0.87) ^*b*^		0.015 ^*a*^ *
Follicular lymphoma (*N* = 16)		0 (0)	0.04 (0.002-0.70) ^*b*^		0.004 ^*a*^ *
MALToma (*N* = 11)		1(8%)	0.12 (0.01–1.20)		0.078 ^*a*^

*Abbreviations*: *CI *confidence interval, *DLBCL *diffuse large B-cell lymphoma, *ENKL *extranodal NK/T-cell lymphoma, *MALToma *mucosa-associated lymphoid tissue lymphoma, *MEITL *monomorphic epitheliotropic intestinal T-cell lymphoma, *ITCL *intestinal T-cell lymphoma

^*a*^ Fisher’s exact test

^*b*^ Haldane-Anscombe correction

* P < 0.05

### Patients’ pathology characteristics

The pathological diagnosis for the 13 MEITL patients was based on endoscopic biopsies in six cases and surgical specimens in seven cases (six following perforation and one following elective resection). CD3 immunopositivity was noted in all (100%). Expression of CD8 and CD56 was noted in 88.9% and 66.7% of patients, while CD 20 was noted in only 8.3%. In addition, CD4, CD5 immunostaining and EBER in situ hybridization were all negative.

### Factors associated with survival

**Fig. 2:**
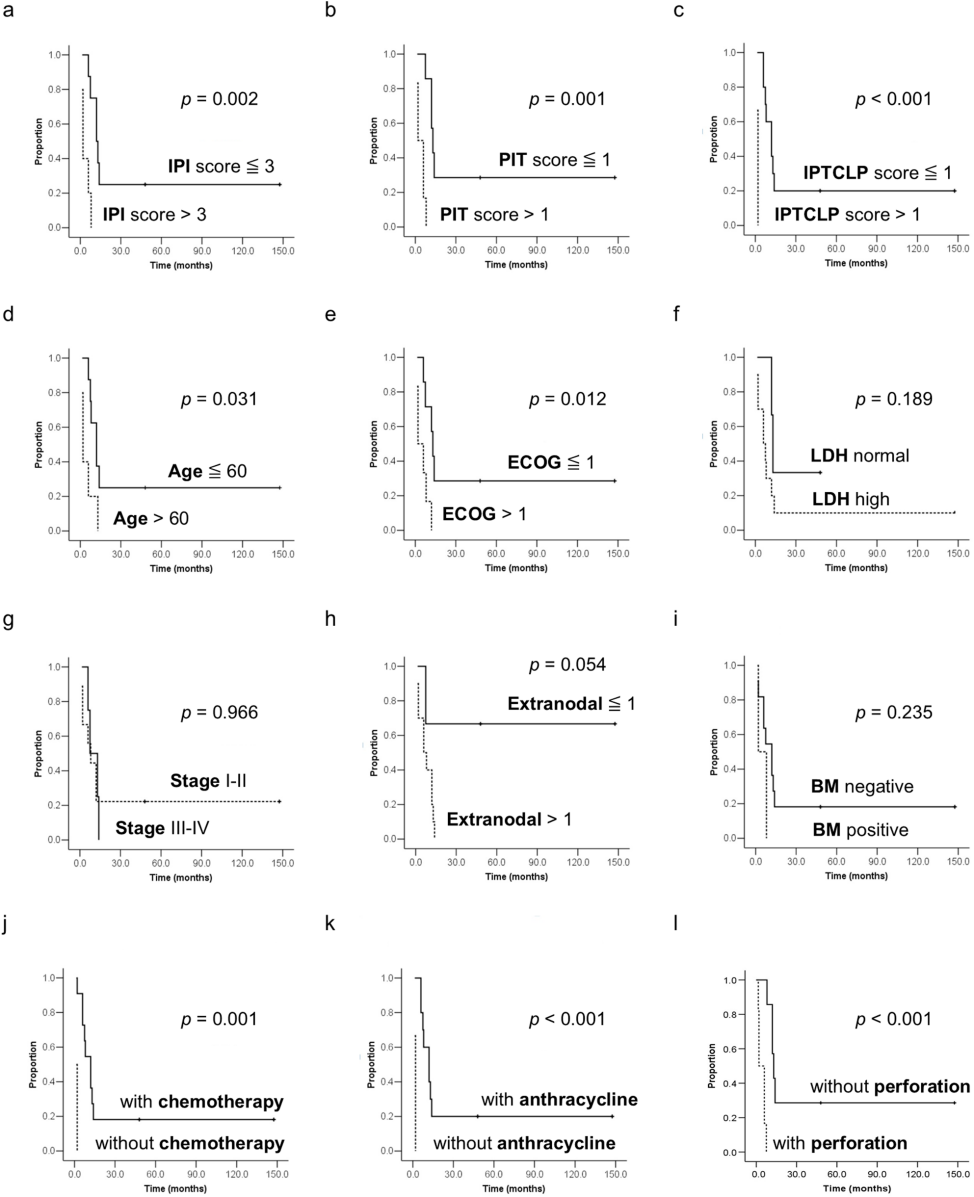
Overall survival of MEITL patients according to different parameters. **a** IPI scores; **b** PIT scores; **c** IPTCLP scores; **d** Age; **e** ECOG performance status; **f** serum LDH level; **g** Ann Arbor stage; **h** Extranodal involvement; **i** Bone marrow involvement; **j** Presence of chemotherapy; **k** Presence of anthracycline therapy; **l** Presence of perforation

We compared the top three small bowel T-cell lymphomas with the most common B-cell lymphoma, DLBCL, by survival analysis with Kaplan-Meier curves. The survival curve of MEITL differed statistically significantly from DLBCL, but not significantly from ITCL or ENKL. There was no median survival of the ITCL as the estimated survival probability did not drop to 50% or below. The median survival of MEITL was 8.0 months, while that of ENKL and DLBCL was 14.0 months and 40.0 months respectively (Fig. 1c). International Prognostic Index (IPI), Prognostic Index for T-cell lymphoma (PIT), and International Peripheral T-Cell Lymphoma Project (IPTCLP) scores all correlated with overall survival in patients with MEITL. Analyzing the effect of individual factors, the overall survival was significantly associated with age, Eastern Cooperative Oncology Group (ECOG) performance status, and perforation. The presence of chemotherapy, especially anthracycline therapy, also correlated with overall survival (Fig. 2).

### Patients’ medical imaging and enteroscopy characteristics

The distribution of these MEITL patients showed jejunum to be the predominant site (Fig. 1d). Jejunal involvement was observed in the majority of MEITL cases (85%). Six cases (46%) involved two small intestinal segments: four involving the duodenum and jejunum, and two involving the jejunum and ileum. There were no significant clinical or prognostic differences between these cases and those with single-segment involvement. This lack of significance is likely attributable to the limited sample size (*N* = 13), which restricts the power of the subgroup analysis.

**Fig. 3:**
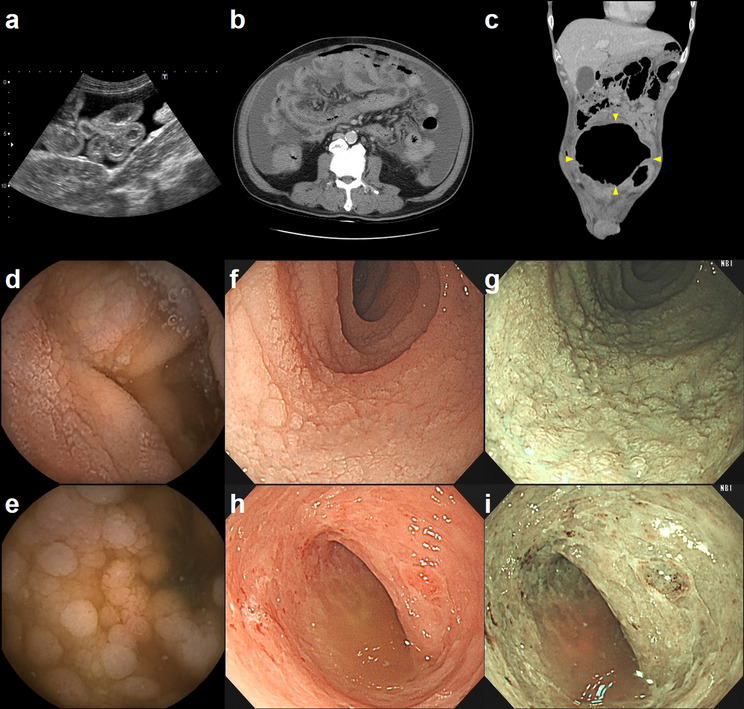
Representative images of MEITL. **a** Abdominal ultrasound image; **b**, **c** Abdominal computed tomography images, arrowheads indicate the location of the cavity; **d**, **e** Capsule endoscopy images; **f**-**i** Single-balloon enteroscopy images: **f**, **g** mosaic mucosal pattern, white light and narrow band imaging; **h**, **i** diffuse erosions with scattered shallow ulcers, white light and narrow-band imaging

Imaging of MEITL frequently reveals infiltrative small bowel lesions, manifesting as diffuse wall thickening and luminal narrowing on abdominal sonography (Fig. 3a) or computed tomography (CT) (Fig. 3b and c). On CT specifically, MEITL appears as either multifocal wall thickening or a cavitary mass. In the context of perforation, pneumoperitoneum was identified in three cases (50%) and ascites in five (83%). An additional three cases exhibited ascites attributed to severe hypoalbuminemia (< 2.0 g/dL). Typical endoscopic findings in MEITL included thickened, edematous mucosa and shallow ulcers. Other frequent observations included the mosaic mucosal pattern (innumerable coarse or fine granular elevations) and diffuse erosions, whereas polypoid features were less common (Table 3). CE findings included diffuse mucosal thickening with white villi or granular elevations (Fig. 3d and e). Similarly, single-balloon enteroscopy effectively visualized these mosaic mucosal patterns (Fig. 3f and g), diffuse erosions, and scattered shallow ulcers within the small bowel (Fig. 3h and i). Narrow-band imaging (NBI) was particularly useful for highlighting these mucosal changes.

**Table 3:** Imaging features of MEITL patients

		Characteristic features	*n* (%)
CT findings (*N* = 13)		Multifocal bowel wall thickening	11 (85%)
		Cavitary mass	2 (15%)
		Pneumoperitoneum	3 (23%)
		Ascites	8 (62%)
Endoscopic findings (*N* = 6)		Thickened / edematous mucosa	6 (100%)
		Shallow ulcers	6 (100%)
		Mosaic mucosal / granular pattern	4 (67%)
		Diffuse erosions	4 (67%)
		Polypoid pattern	3 (50%)

### Patients’ treatment strategies and related outcomes

Most patients died within one year after diagnosis. Two MEITL patients remained in CR status on reviewing, and the other 11 patients had passed away. Most MEITL patients had received chemotherapy (85%), especially CHOP-based regimen as first line (62%), while the rest received best supportive care alone due to poor performance status. Four patients reached complete remission (CR) after chemotherapy. One of the four patients relapsed soon and still passed away after changing to second-line chemotherapy. Two patients receiving chemotherapy along with autologous stem cell transplantation (ASCT) reached CR. One remained in CR for 5 years, the other relapsed later at lung and died of fungal infection with disease free survival of 4.5 months. The last one of the four patients did not receive ASCT because of difficulty in collecting enough peripheral blood stem cells himself and no suitable donor for allogeneic stem cell transplantation. This patient relapsed later but remained in CR for more than 10 years after the second-line chemotherapy regimen (ESHAP).

## Discussion

The most common histopathology types of small bowel malignancies include adenocarcinoma, lymphoma, sarcoma, and neuroendocrine tumor/carcinoid tumor, ranking varied across different regions. Neuroendocrine tumor/carcinoid tumor and adenocarcinoma are the most common small bowel malignancies in the United States [8], while lymphoma and gastrointestinal stromal tumor (GIST) are the most common types in Taiwan [4]. Among small bowel malignancies, lymphoma is a heterogeneous group of malignancies affecting the immune system. Primary small bowel lymphoma includes several different kinds of histological subtypes, such as DLBCL, MALToma, EATL, MEITL, mantle cell lymphoma, Burkitt lymphoma, follicular lymphoma, immunoproliferative small bowel disease, ITCL, and ENKL [9]. B-cell lymphomas account for a higher percentage in comparison with T-cell lymphomas. Male gender, older than 75 years of age, and non-white racial groups are associated with a worse prognosis in all patients with small bowel lymphoma [8]. The prognosis of T-cell lymphoma is generally poor except for indolent T-cell lymphoproliferative disorders of the gastrointestinal tract [10].

MEITL, a rare primary T-cell lymphoma of the gastrointestinal tract, is thought to originate from the intraepithelial T-cells with frequent γδ T-cell receptor expression [11], and increased CD8αα expression [12, 13]. MEITL typically localizes to the small bowel (jejunum more frequent than duodenum and ileum), and rarely to the colon or stomach. Patients with MEITL are usually of older age (median age: 58–67 years) and male predominance, and usually present with abdominal pain, weight loss, and chronic diarrhea. The prognosis is usually poor with a median survival of 7–15 months [6, 11–16]. Our case series showed similar clinical presentations, but some MEITL patients presented with weight gain rather than weight loss after hypoalbuminemia-related generalized edema. In addition, we found a significantly higher bowel perforation rate in MEITL patients than in non-MEITL small bowel lymphoma patients. The fact that all perforations in the MEITL group occurred at the time of initial diagnosis suggests these events are a manifestation of disease biology rather than a consequence of treatment. Beyond its comparison to indolent lymphomas (e.g., follicular lymphoma), MEITL demonstrated a perforation frequency that surpassed even that of other aggressively lymphomas, such as DLBCL and mantle cell lymphoma. Bowel perforation led to an inferior outcome in MEITL patients (Fig. 2l), similar to other gastrointestinal lymphoma patients [17]. A higher incidence of perforation has been noted in intestinal T-cell in comparison to B-cell lymphomas [18]. Both the location factor (small bowel) relating to the bowel wall thickness and histology factor (T cell lymphoma) contribute to the higher risk of perforation [17, 19].

Though the final diagnosis of MEITL is based on histopathology, a gastroenterologist should have this disease entity in his differential diagnosis after obtaining typical image information. Cross-sectional imaging studies including CT and magnetic resonance imaging (MRI) are the best noninvasive tools for diagnosing, evaluating, and staging small bowel cancer. For small bowel lymphoma, the most commonly recognized radiologic patterns include infiltrative/pseudoaneurysmal, polypoid, endoexoenteric/cavitary, mesenteric and stenosing forms [20]. The infiltrative form, the most common small bowel lymphoma form, presents with bowel wall thickening, distortion or loss of the fold pattern, nodularity, and either luminal narrowing or aneurysmal dilatation [21]. In our patients, infiltrative form (85%) is the most common CT presentation of the MEITL (Fig. 3b). The endoexoenteric/cavitary form appears as a large soft-tissue mass that communicates with the bowel lumen and appears cavitary, representing a sealed-off localized perforation of bowel into the soft-tissue mass in the mesenteric space. The endoexoenteric/cavitary form accounts for the rest (15%) of our MEITL patients (Fig. 3c), corresponding to the fact that perforation is common in MEITL. The stenosing form is not present in our patients, but has been reported in the advanced stage after recurrence [22].

In addition to cross-sectional imaging studies, a gastroenterologist may use various endoscopic tools for evaluation of MEITL. The characteristic endoscopic features of MEITL include diffuse mucosal thickening and edema with multiple shallow ulcers, and a nodular or mosaic mucosal pattern [23, 24]. The mucosal granular change is more prominent under NBI (Fig. 3g and i) or indigo carmine chromoendoscopy [25]. A circumferential ulcer with stenosis may be present in the advanced stage [22]. While CE is a valuable diagnostic tool, it is limited by potential device retention, the risk of overlooking proximal small bowel tumors, and the inability to perform tissue biopsy [10]. In cases where imaging reveals suspected small bowel tumors, DAE is the preferred diagnostic modality because it avoids the complication of capsule retention and allows for direct biopsy. DAE provides a histological diagnostic yield of 60–80% for small bowel tumors [26]. In comparison with DAE, CE has a higher rate of complete examination of the small bowel and is less invasive. Small bowel CE is the currently recommended survey tool for patients with an increased risk of a small bowel tumor [27]. Deep learning-based artificial intelligence (AI) can detect small bowel lesions on CE images more sensitively and rapidly than conventional gastroenterologist reviews [28]. Recent studies highlighting AI’s improved diagnostic accuracy in small bowel lymphoma [29, 30] suggest that these tools could soon become a standard diagnostic approach for MEITL.

Notably, we identified two common laboratory markers—WBC counts and serum albumin levels—that differed significantly between MEITL and non-MEITL cases within our overall cohort of small bowel lymphoma patients. Clinical evidence frequently associates small bowel-perforated MEITL with leukocytosis [31, 32]. In our cohort, hemograms revealed significantly higher WBC counts among MEITL patients, likely secondary to the high prevalence of complications such as bowel perforation and secondary infection. Although small bowel perforation in DLBCL can also manifest with elevated WBC counts [33], the significantly lower incidence of perforation in that group accounts for the lower median WBC levels compared to the MEITL group. Consequently, leukocytosis may serve as a characteristic diagnostic indicator for MEITL in the differential diagnosis of small bowel lymphomas. Biochemical analysis revealed significant hypoalbuminemia in MEITL patients, likely resulting from protein-losing enteropathy (PLE) secondary to MEITL-induced mucosal damage. Consequently, low albumin levels may serve as a clinical indicator of MEITL in cases of small bowel lymphoma. For patients presenting with chronic diarrhea and hypoalbuminemia, MEITL should be considered earlier in the differential diagnosis. In this study, one patient’s critically low albumin (1.5 g/dL) coincided with an alpha-1 antitrypsin clearance exceeding 10,000 mL/day, confirming PLE. Notably, albumin levels did not correlate with the frequency or duration of diarrhea. This discrepancy suggests that chronic diarrhea is an unreliable marker of severity, as PLE patients can maintain formed stools despite significant protein loss.

The International Prognostic Index (IPI) was originally developed to predict outcomes in aggressive non-Hodgkin lymphoma [34]. In the present study, several clinical prognostic models—including the IPI (score ≤ 3), PIT (score ≤ 1), and IPTCLP (score ≤ 1)—effectively predicted superior outcomes for MEITL patients. Since age and performance status are primary indicators of a patient’s capacity to tolerate intensive therapy, younger age (≤ 60) and favorable performance status (ECOG ≤ 1) correlated significantly with prolonged overall survival. While anthracycline-based chemotherapy has been shown to offer superior survival rates compared to surgery or radiotherapy alone [35, 36], our findings confirm that MEITL patients receiving any chemotherapy, particularly anthracycline-based regimens, achieve significantly better outcomes than those who do not. However, these results may be subject to selection bias, as systemic therapy is typically reserved for patients with fewer comorbidities and higher performance status. Furthermore, the occurrence of bowel perforation—which often precludes the administration of systemic therapy—was strongly associated with poor survival. These observations underscore the aggressive clinical course of MEITL and highlight the urgent need for novel, intensive therapeutic strategies.

Because conventional surgery, radiotherapy, and anthracycline-based chemotherapy typically yield disappointing results, a definitive standard of care for MEITL has yet to be established. However, the IVE/MTX (ifosfamide, etoposide, epirubicin/methotrexate) protocol has emerged as a superior first-line therapy due to its enhanced tumor control and manageable toxicity [36]. For transplant-eligible candidates, the preferred strategy is induction chemotherapy followed by ASCT, which can facilitate long-term remission if performed during the first complete or partial remission [7, 37]. Our study reinforces this approach: of the two patients treated with the IVE/MTX-ASCT sequence, one achieved an overall survival (OS) exceeding 48 months, while the other relapsed at three months with an OS of 14 months. Furthermore, proactive nutritional support via parenteral or enteral feeding is recommended to enhance performance status and treatment tolerance [34]. Given these findings, a comprehensive strategy combining an effective chemotherapy regimen like IVE/MTX, consolidative ASCT, and proactive nutritional support currently represents the most viable therapeutic option for MEITL.

There are certain limitations in our study. First, the single center study design ensured a high degree of care protocol uniformity and data granularity, but it might limit the generalizability of our findings. The results should be interpreted cautiously when applying them to different healthcare settings or geographic regions. Second, the relatively small sample size might limit the statistical power to detect subtle differences between groups. More cases would be necessary for both confirmation of our preliminary findings and further subgroup analysis. Third, the retrospective nature of this study might introduce inherent risks of selection and information bias, as we relied on existing medical records that were not designed for research purposes originally.

## Conclusions

MEITL is a rare primary gastrointestinal lymphoma characterized by a poor prognosis and a higher prevalence among Asian populations. Clinicians should maintain a high index of suspicion for MEITL in Asian patients presenting with jejunal lesions, weight loss, chronic diarrhea, and bowel perforation, particularly when accompanied by characteristic imaging and endoscopic findings. Early diagnosis is critical, as it allows for the maintenance of superior nutritional status; furthermore, proactive nutritional support before and during treatment may reduce morbidity and mortality. Currently, intensive chemotherapy followed by ASCT appears to be the preferred therapeutic strategy. 

## Data Availability

All data generated or analyzed during this study are included in this published article.

## Abbreviations

AI: Artificial intelligence
ASCT: Autologous stem cell transplantation
CE: Capsule endoscopy
CR: Complete remission
CT: Computed tomography
DAE: Device-assisted enteroscopy
DLBCL: Diffuse large B-cell lymphoma
EATL: Enteropathy associated T-cell lymphoma
ECOG: Eastern Cooperative Oncology Group
ENKL: Extranodal NK/T-cell lymphoma
GIST: Gastrointestinal stromal tumor
IPI: International Prognostic Index
IPTCLP: International Peripheral T-Cell Lymphoma Project
MALToma: Mucosa-associated lymphoid tissue lymphoma
MEITL: Monomorphic epitheliotropic intestinal T-cell lymphoma
MTX: Methotrexate
MRI: Magnetic resonance imaging
NBI: Narrow-band imaging
PIT: Prognostic Index for T-cell lymphoma
PLE: Protein-losing enteropathy
ITCL: Intestinal T-cell lymphoma
